# *Chrysanthemum × grandiflora* leaf and root transcript profiling in response to salinity stress

**DOI:** 10.1186/s12870-022-03612-x

**Published:** 2022-05-12

**Authors:** He Liu, Yu Liu, Ning Xu, Ying Sun, Qiang Li, Liran Yue, Yunwei Zhou, Miao He

**Affiliations:** 1 College of Landscape Architecture, Northeast Forestry University, No. 26 Hexing Road, Harbin, 150006 Heilongjiang China; 2 College of Horticulture, Jilin Agricultural University, 2888 Xincheng Street, Changchun, 130118 Jilin China

**Keywords:** Salt stress, Osmotic adjustment, Transcriptome, Chrysanthemum, DEGs, Transcription factors

## Abstract

As high soil salinity threatens the growth and development of plants, understanding the mechanism of plants’ salt tolerance is critical. The *Chrysanthemum × grandiflora* is a newly developed species with a strong salt resistance that possesses multiple genes controlling its quantitative salt resistance. Because of this multigene control, we chose to investigate the plant stress genes overall responses at the transcriptome level. *C. grandiflora* were treated with a 200 mM NaCl solution for 12 h to study its effect on the roots and leaves via Illumina RNA sequencing. *PAL*, *CYP73A*, and *4CL* in the phenylpropanoid biosynthesis pathway were upregulated in roots and leaves. In the salicylic acid signal transduction pathway, *TGA7* was upregulated in the roots and leaves, while in the jasmonic acid signal transduction pathway, *TIFY9* was upregulated in the roots and leaves. In the ion transporter gene, we identified *HKT1* that showed identical expression patterns in the roots and leaves. The impact of NaCl imposition for 12 h was largely due to osmotic effect of salinity on *C. grandiflora*, and most likely the transcript abundance changes in this study were due to the osmotic effect. In order to verify the accuracy of the Illumina sequencing data, we selected 16 DEGs for transcription polymerase chain reaction (qRT-PCR) analysis. qRT-PCR and transcriptome sequencing analysis revealed that the transcriptome sequencing results were reliable.

**Supplementary Information:**

The online version contains supplementary material available at 10.1186/s12870-022-03612-x.

## Introduction

Soil salinity is a serious global threat to the environment and to agricultural production. Currently, more than 20% of the world’s arable land (1 billion hectares) is affected by salinity, and this number is increasing due to global climate change and poor irrigation and fertilization management. Generally, when the salt content of the soil exceeds 1%, it becomes difficult for plants to grow. Salt stress affects the various stages of plant seed germination, growth, differentiation, and development. The study of plant salt tolerance and the development of salt-tolerant plant varieties are critical for environmentally sustainable development [1, 2]. At present, salt tolerance and the potential mechanisms of salt tolerance of a large number of plant varieties have been studied, such as *Arabidopsis* [3, 4], tobacco [5], corn [6] and tomato [7]. Research on soybeans [8] and rice [9] have shown that the method of increasing the plant expression of intrinsic salt tolerance genes can effectively enhance their salt tolerance.

Salinity stress has a significant impact on plant growth and development. First, salt stress reduces plant water absorption capacity and inhibits their growth. This is called osmotic stress, in which lowering the soil solution water potential by high level of salts induces an early osmotic stress, whereas ionic imbalance and toxicity occurs after a longer time of stress [10]. If excessive salt enters the transpiration stream of plants, it damages cells in the leaves, thus further affecting plant growth. When the salt level reaches a threshold value, the plant cannot maintain an ion balance, which can also cause secondary reactions such as oxidative stress [11]. Studies have shown that under salt stress conditions, more transcriptome changes were observed in the root tissue than in the leaf tissue of the Chrysanthemum cultivar ‘Jinba’ [12]. Its response to stress was mainly manifested in the downregulation of genes in the leaves and roots. Under drought conditions and salt stress, transcriptome changes were observed more in the leaf tissue compared with the root tissue of *Chrysopogon zizanioides*. The response to stress in the transcriptome of *C. zizanioides* was mainly manifested in the upregulation of genes in the leaves and roots [13]. Finally, when comparing root with leaf tissues in *Rosa chinensis* under drought conditions, the transcriptome underwent more changes in the leaf tissues, with a downregulation of genes in the leaves and roots [14].

Generally, plants respond to salt stress by developing a series of morphological, physiological, biochemical, and molecular regulation mechanisms to ensure normal growth and development. These mechanisms include osmotic regulation, ion homeostasis, signal transduction, and the induction of antioxidant enzyme activity. Salt tolerance is a complex trait controlled by genetic factors. The functions of many salt-responsive genes involve the regulation of ion accumulation, stress signal transduction, transcription regulation, redox reactions, and the accumulation of specific osmolytes [15]. Transgenic plants with different degrees of enhanced salt tolerance can be obtained by controlling the expression of some salt tolerance genes. These genes include HKT (high affinity potassium transporter) [16], AKT (K^+^ channel gene) [17], NHX (Na^+^/ H^+^ antiporter gene) [18], WRKY transcription factor gene [19], NAC transcription factor gene [20], bZIP transcription factor gene [21], and ERF transcription factor gene [22].

The *C. grandiflora* is a new ground-planted chrysanthemum species selected after natural hybridization and satellite loading based on the introduction of ground cover chrysanthemums (*Chrysanthemum morifolium*). *C. morifolium* characterized by its long flowering period, copious dense flowers, bright color, as well as high resistance to stress. This new species has become a popular urban green ground cover plant in recent years [23]. Transcriptome sequencing was used to find *C. grandiflora* genes that were resistant to abiotic stress, and thus provides a physical foundation to improve plant stress resistance and to provide new stress-resistant materials; both would have a great significance in the study of chrysanthemum germplasm resources. Current studies have shown that when the chrysanthemum is exposed to salt stress, genes encoding proteins related to osmotic regulation, ion transport (Na^+^, K^+^, and Ca^2+^ transport), ROS scavenging, and ABA signaling are all affected in the roots and leaves [12]. Osmotic regulation genes and Ca^2+^ transport genes overlap in the roots and leaves after salt treatment, which may serve as the main regulator of the plant salt response. These results indicate that the regulation of the transcriptome plays a key role in the morphological and physiological adaptation of chrysanthemum roots and leaves in response to salt stress [12]. In our research, we discovered the key metabolic pathways, a large number of salt-tolerant genes, and the underlying mechanisms of adaptation of *C. grandiflora* in response to salt stress.

## Materials and methods

### Plant materials and experimental design

*Chrysanthemum×grandiflora* is a new chrysanthemum variety cultivated in Northeast Forestry University. It is a new ground-grown chrysanthemum variety group based on the introduction of *Chrysanthemum morifolium*, after natural hybridization and satellite loading. The stem explants collected from the mother plant were rinsed in tap water for 60 min. The stems were sterilized with 75% ethanol solution for 30 s, rinsed with sterile distilled water for 1 min, and soaked in a 4% sodium hypochlorite solution for 10 min. The stems were then rinsed with sterile, distilled water 5 times, placed on sterile filter paper to absorb the water, and cut open into sections about 2.5 cm long. One or more of the shoots were inoculated in MS medium. When the plant had grown 7–8 cotyledons, the seedlings were transplanted into plastic pots with a diameter of 10 cm, a bottom diameter of 7.3 cm, and a height of 8.5 cm, and the cultivation substrate was humus: vermiculite: perlite (2:1:1, v/v/v), pH 6.65 [24].

In our previous study, it was found that when *C. grandiflora* was treated with 200 mM NaCl solution concentration, and compared with the control group in the normal growth environment, the treated plants showed an obvious stress response, such as changes in plant morphology, antioxidant enzyme systems, and ion accumulation, and therefore this concentration was used in the current work. When the plants had grown 9–10 leaves, they were treated with 200 mM NaCl. We irrigated with 60 mL of 200 mM NaCl at 8 am. After 12 h of treatment, all the root and leaf samples of the treated and the control groups were taken for transcriptome sequencing. Root and leaf samples were collected at 0, 0.5, 3, 6, and 9 d after plant cultivation to determine the relative water content of leaves, Na^+^, K^+^, and H_2_O_2_ content and SOD, POD, and CAT activity.

The plant growth media contained 30 g/L sucrose and 0.6% (w/v) agar and was adjusted to pH 5.8–6.0 and autoclaved at 121 °C for 20 min. The plants were grown in plant culture pots (11 cm high × 7 cm diameter, 300 mL) and placed in an air-conditioned incubator with a temperature of 25 °C ± 2 °C, a relative humidity of 50–70% and a G13 fluorescent lamp (Philips, Tianjin, China) as a light source, the light intensity was 75 μmol m^− 2^ s^− 1^.

### Determination of antioxidant enzyme activity and H_2_O_2_ content

From the roots and leaves, 0.1 g fresh weight were taken and the antioxidant enzyme activity was measured on 0, 0.5, 3, 6 and 9 d. The activities of SOD (U/g FW), POD (U/g FW), and CAT (nmol/min/g FW), and the content of H_2_O_2_ (μmol/g FW) were determined according to the instructions included in the chemical analysis kit (Solebao Technology Co., Ltd., Beijing, China). Experimental methods are available at https://www.solarbio.com/. One-way anova analysis of variance was performed, and then Dunnett’s test in SPSS26 was used to compare the means of all treatments..

### Determination of Na^+^ and K^+^ content

The collected root and leaf samples were dried at 80 °C for 2 d. The samples were then ground into powder, and 1 mM HCl was added to the samples to react for 12 h. The volume of the filtered solution was adjusted to 50 mL, and the content of Na^+^ and K^+^ was measured using 4210MP-AES (Agilent Technology, USA) [25].

### Transcriptome sequence annotation

Based on the original data, we calculated the proportion of unknown nucleotides, and the base (Q20) base recognition accuracy exceeded 99.0%. In order to annotate the reassembled sequence, after cleaning the original reader and processing the poor-quality reader, the assembled transcript was aligned with NCBI (https://www.ncbi.nlm.nih.gov/; June 2021, 1st visit).

### Gene expression level analysis

FPKM was used to calculate gene expression levels through density distribution and analysis of the number of selected transcripts, and the expression of all the samples was analyzed. According to the results of FPKM, the correlation between the samples was calculated to determine the stability and reliability of the experimental operation.

### Identification and functional annotation of DEGs

The DEGs were mapped to each item in the GO database. The hypergeometric test method was used to detect significantly rich GO annotations. The corrected *P* value was < 0.05. In addition, the hypergeometric test calculated the number of DEGs at different levels to determine the main pathways involved in salt tolerance. When *P* < 0.05, the KEGG pathway was significantly enriched [26, 27].

### qRT-PCR analysis

For qRT-PCR analysis in the KEGG enrichment pathway, 16 candidate genes, including 8 upregulated genes and 8 downregulated genes, were randomly selected. The Roche LightCycler96® system was used for qRT-PCR. The differential expression analysis of each candidate gene used the 2^-△△CT^ method, and each candidate gene was repeated three times [28].

## Results

### Effects of salt stress on the contents of Na^+^ and K^+^ and the activities of antioxidant-related enzymes in the roots and leaves of *C. grandiflora*

**Fig. 1:**
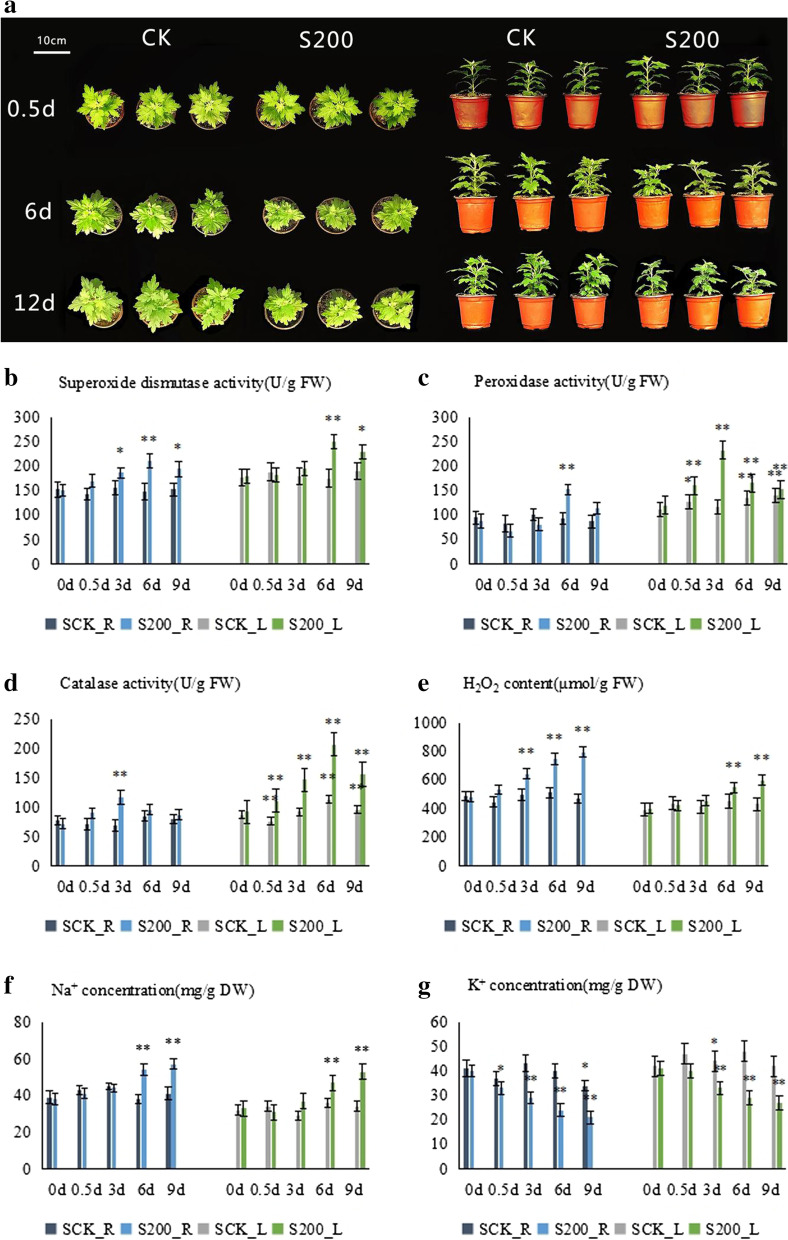
The effect of 200 mM NaCl exposure on the growth, enzymatic antioxidant activities, ion and H_2_O_2_ contents of *C. grandiflora*: (**a**) growth phenotype, (**b**) SOD, (**c**) POD, (**d**) CAT, (**e**) H_2_O_2_, (**f**) Na^+^, (**g**) and K^+^. ** (*P* < 0.01) represents highly significant differences and * (*P* < 0.05) represents significant differences for the specified treatment based on Dunnett’s test

After treatment with 200 mM NaCl solution, the growth of *C. grandiflora* in the control group was significantly better than the growth of those in the treatment group (Fig. 1a). As the treatment time increased, the relative water content of leaves gradually decreased (Table 1). The activity of superoxide dismutase (SOD) in the roots and leaves increased initially and then decreased (Fig. 1b). The SOD activity of the roots and leaves on the sixth day of treatment was significantly higher than the activity at the other time points. The peroxidase (POD) activity in the roots and leaves (Fig. 1c) showed a trend of an initial increase followed by a subsequent decrease. The POD activity of the roots was significantly higher at Day 6 of treatment than at the other time points, and the activity of the leaves was significantly higher than at the other time points at Day 3 of treatment. CAT activity in the roots and leaves showed a trend of an initial increase followed by a subsequent decrease similar to that of POD. CAT activity in the roots at Day 3 and in the leaves at Day 6 were significantly higher than at the other time points in the treatment (Fig. 1d). The H_2_O_2_ content in both the roots and the leaves increased over the course of treatment with the most significant content change occurring after Day 3 in the roots and after Day 9 in the leaves (Fig. 1e). The Na^+^ content in both the leaves and the roots increased as well with the greatest Na^+^ change occurring in the roots and the leaves at Day 9 of treatment (Fig. 1f). The K^+^ content showed a downward trend in both the roots and the leaves with the most significant change in K^+^ concentration occurring after Day 3 for both the leaves and the roots (Fig. 1g). Under salt stress, antioxidant enzyme activities and ion accumulation in the roots and leaves of *C. grandiflora* began to change after 12 h of 200 mM NaCl treatment, indicating that the expression of salt stress-responsive genes were upregulated. Therefore, we used RNA-seq to analyze the root and leaf samples of *C. grandiflora* during the 12 h salt stress treatment.

**Table 1:** Relative water content of leaves of *C. grandiflora* leaves under 200 mM NaCl stress. The data are the mean ± standard error

Salt stress days(d)	Control leaf water content(%)	Treatment group leaf water content(%)
0	93.73 ± 0.33	93.71 ± 0.35
0.5	93.65 ± 0.37	93.42 ± 0.43
3	93.51 ± 0.41	92.76 ± 0.45
6	92.47 ± 0.32	90.47 ± 0.36
9	92.28 ± 0.44	87.58 ± 0.38

### Assembly and transcriptome quality assessment

Twelve samples were sequenced on the Illumina sequencing platform. Among them, there were 558,054,470 original fragments. By removing low-quality areas and adapters, 543,328,476 clean readings remained, Q20 > 97.05% (Table S1). Figure S1 shows the transcriptome’s quality and single gene length distribution.

### Transcriptome annotation

Using BLAST to screen data with an *E* value of <1e-5, there were 31,181 (79.57%) unigenes matching the known genes in the NR database, and 21,333 (54.44%) unigenes matching the Swiss-Prot database (Table S2). The sequence with the highest annotation rate in the NR database (*E* value <1e-45) was 32.70% (Fig. S2a). Approximately 70.54% of the unigenes were similar to the identified sequence (Fig. S2b). Among the following 6 species, about 94.30% of the single gene annotations matched *C. grandiflora*, including mugwort (79.55%), sunflower (4.97%), thistle (4.75%), lettuce (3.46%), chrysanthemum (1.08%), and grapes (0.42%) (Fig. S2c).

### Unigenes’ EggNOG functional categories annotation

**Fig. 2:**
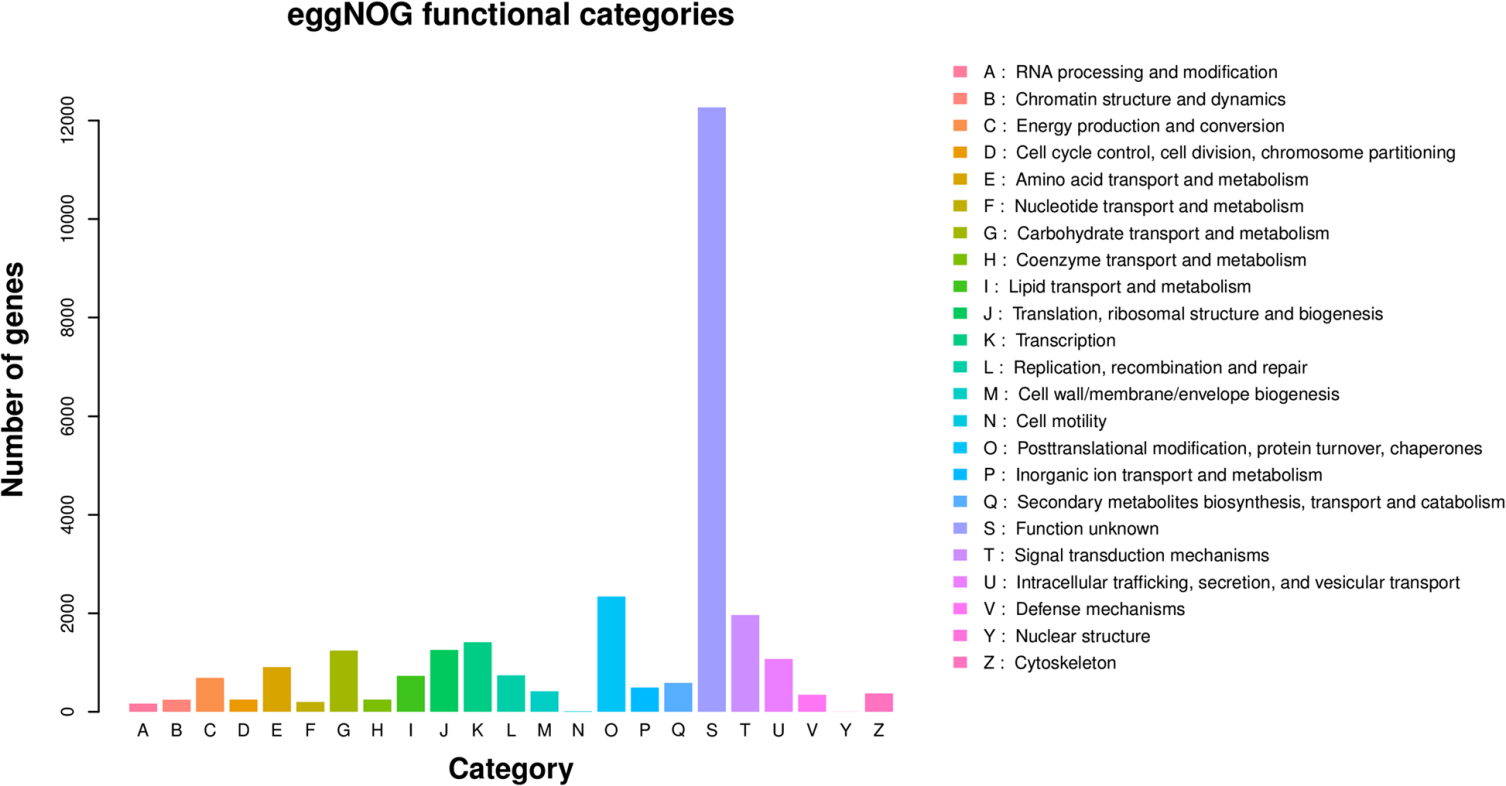
EggNOG functional classification of all unigenes in *C. grandiflora*

Comparing the unigenes with the eggNOG database, we found that 28,580 unigenes in *C. grandiflora* could be classified into 24 categories according to the prediction function (Fig. 2). Unknown function is the most annotated category among these eggNOG categories, followed by post-translational modification, protein renewal, chaperone protein, and nuclear structure in that order.

### Gene expression and differentially expressed gene analysis

**Fig. 3:**
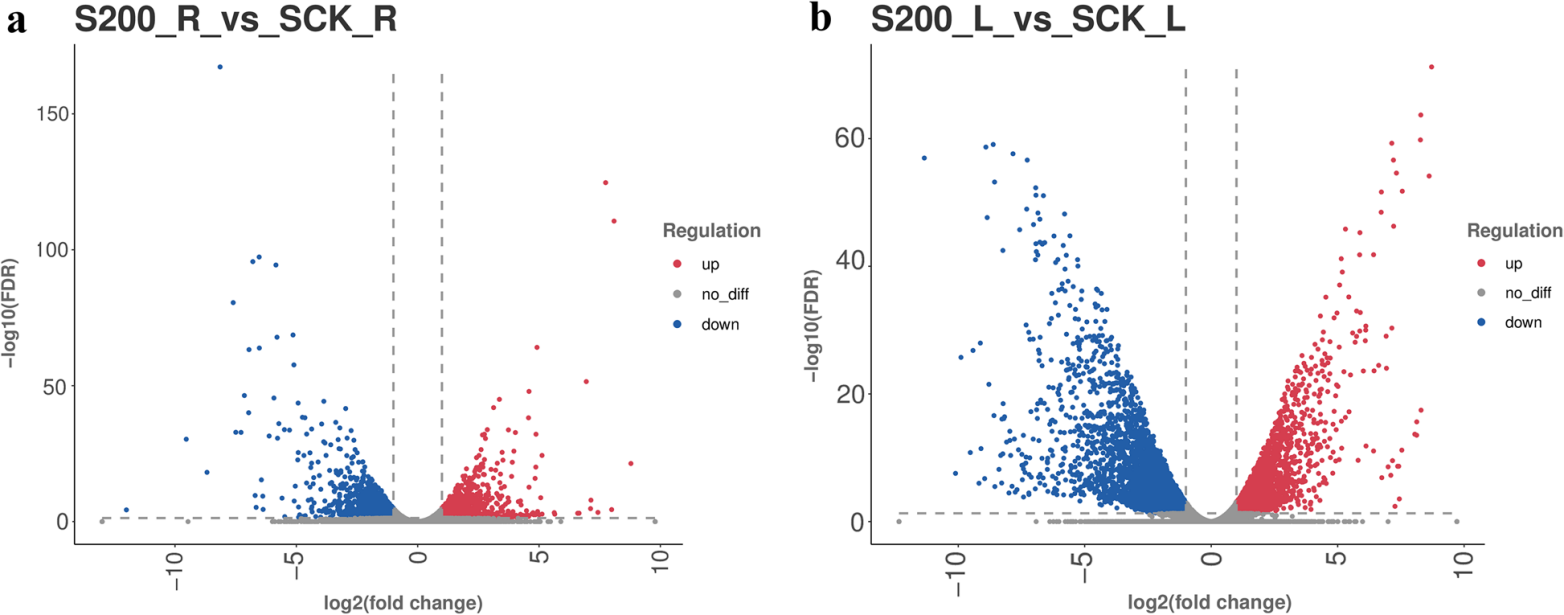
Differentially expressed gene (DEG) expression analysis: (**a**) A volcano map of DEGs in S200_R vs SCK_R, and (**b**) A volcano map of DEGs in S200_L vs SCK_L

**Fig. 4:**
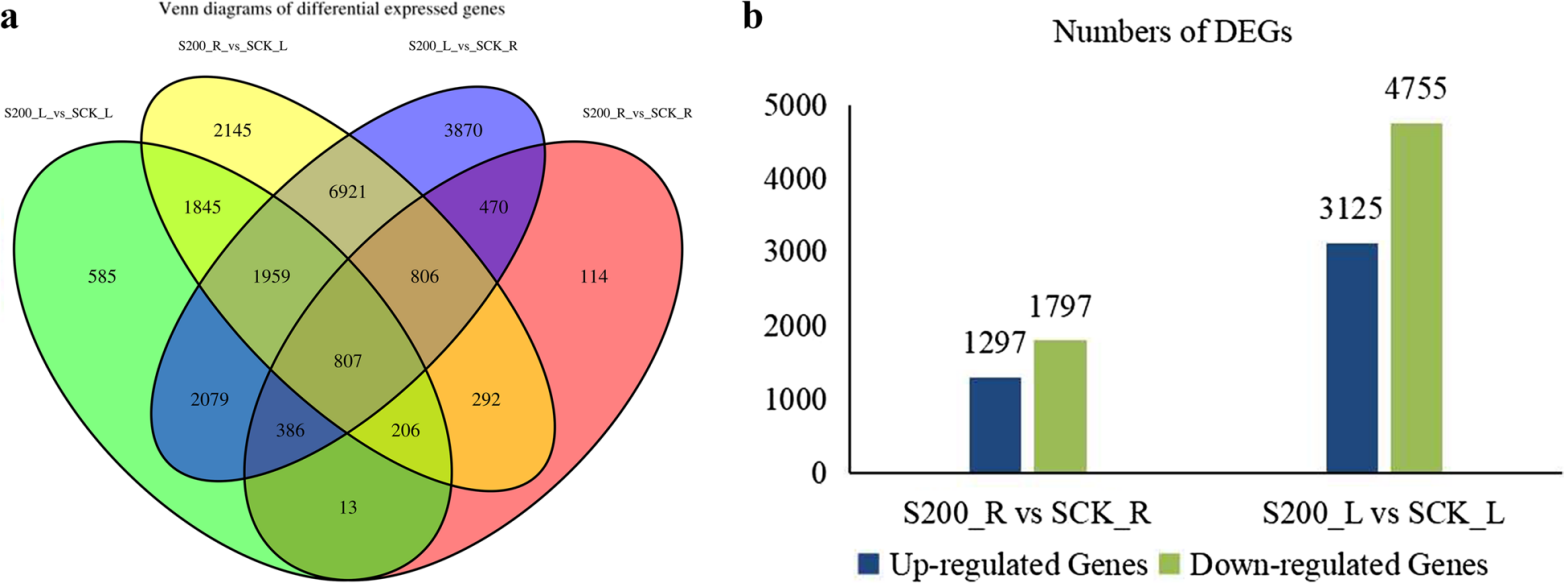
Venn diagram analysis of the number of DEGs after 12 h of NaCl treatment: (**a**) Venn diagram analysis of DEGs annotated in the NR database, and (**b**) the number of DEGs upregulated and downregulated after 12 h of 200 mM NaCl imposition

According to the hierarchical clustering analysis of the differentially expressed gene expression pattern, we found that there were more downregulated expressed genes in the samples of *C. grandiflora* (Fig. 3). Comparing the treatment group with the control group, there were more DEGs in the leaves (Fig. 4a), and the ratio of DEGs in the NR database was 79.57%. A total of 3094 DEGs were obtained from the roots treated for 12 h, and 7880 DEGs were obtained from the leaves treated for 12 h (Fig. 4b). In the roots, there were 1297 upregulated genes, while in the leaves there were upregulated genes.

### DEGs GO annotation enrichment analysis

**Fig. 5:**
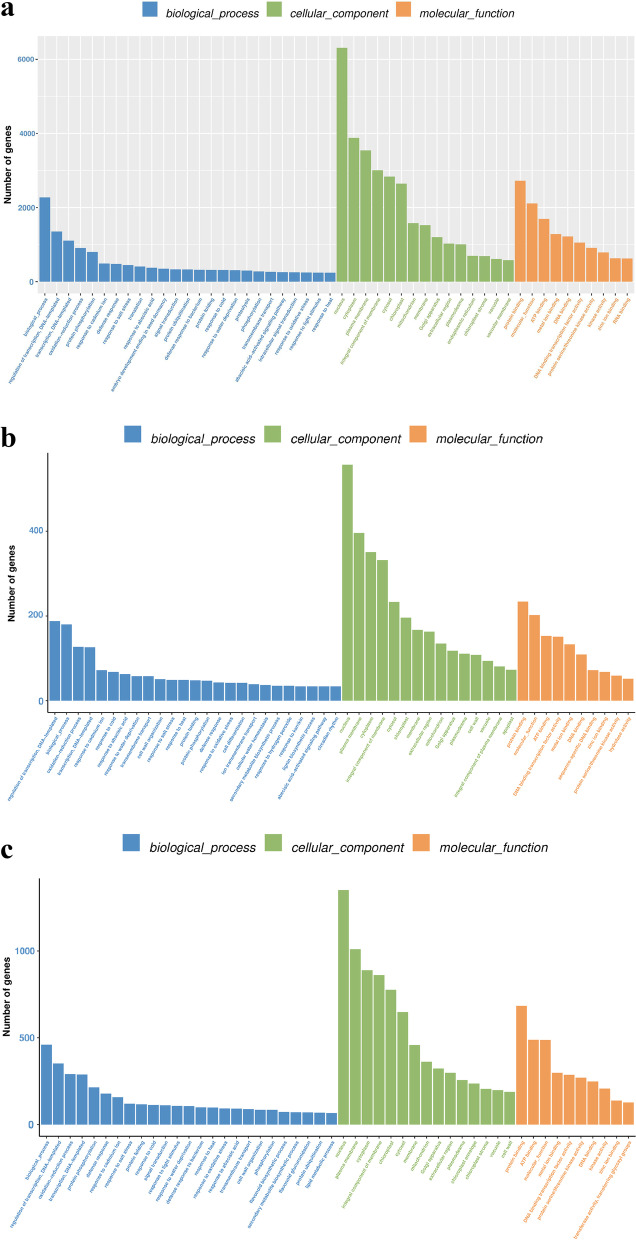
Classification of unigenes of GO annotation of *C. grandiflora*: (**a**) S200_R vs SCK_R vs S200_L vs SCK_L, (**b**) S200_R vs SCK_R, and (**c**) S200_L vs SCK_L

There was a total of 135,710 genes annotated in the GO database in the *C. grandiflora* library undergoing salt stress for 12 h. Among them, there were 49,890 unigenes for biological processes; 45,951 unigenes for cell components; and 39,869 unigenes for molecular functions, including nuclear, cytoplasmic, and cytoplasmic genes. The number of DEGs enriched in membranes, membrane components, and the cytosol was the largest (Fig. 5a). A total of 13,245 genes in the root library were annotated in the GO database. Within these genes, there were 5116 unigenes for biological processes; 4159 unigenes for cell components; and 3970 unigenes for molecular functions, including nuclear, plasma membrane, cytoplasm, and membrane components. The number of DEGs enriched in protein binding was the largest (Fig. 5b). There were 33,729 genes in Ye’s library annotated in the GO database. Among them, there were 12,524 unigenes for biological processes; 11,271 unigenes for cell components; and 9934 unigenes for molecular functions, including nuclear, plasma membrane, cytoplasm, and membrane components. The number of DEGs enriched in chloroplasts was the largest (Fig. 5c).

### Enrichment analysis of the KEGG pathway of DEGs

**Fig. 6:**
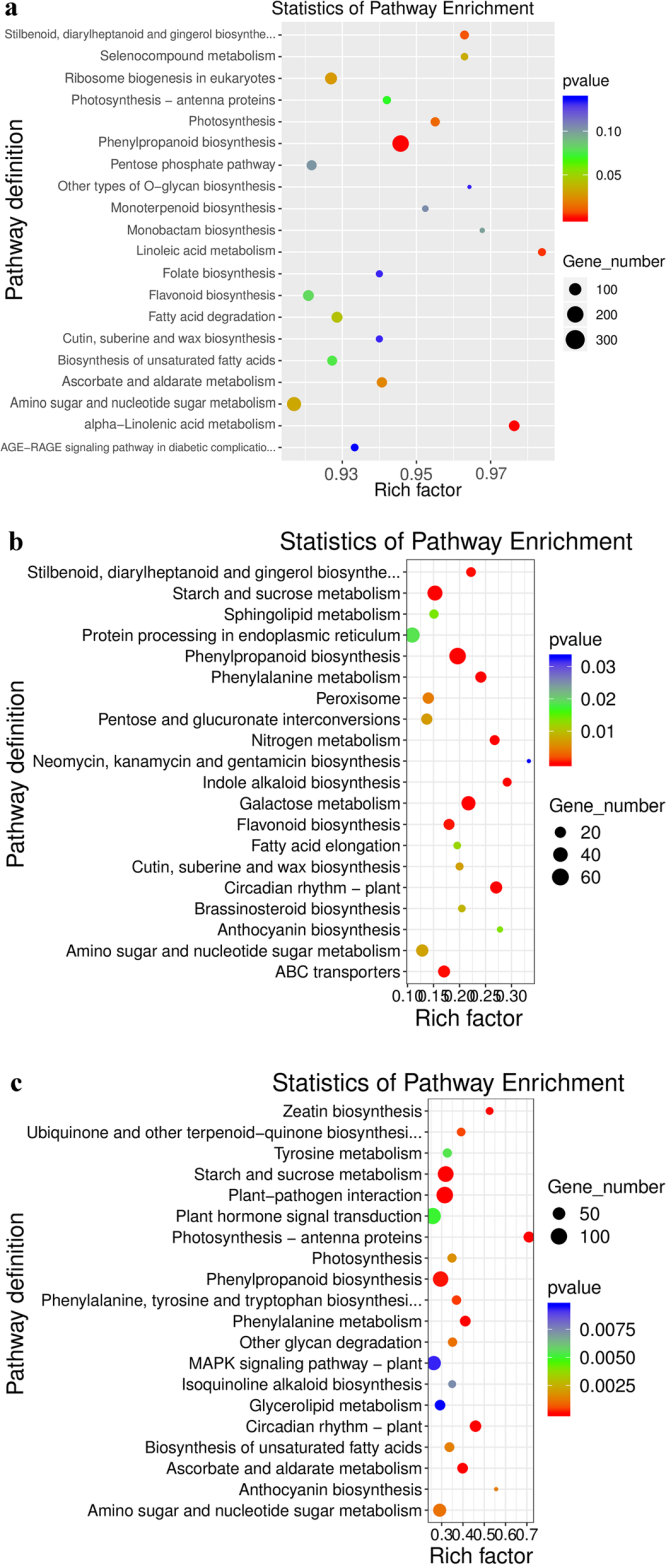
Enrichment analysis of the KEGG pathway of DEGs in *C. grandiflora*: (**a**) S200_R vs SCK_R vs S200_L vs SCK_L, (**b**) S200_R vs SCK_R, and (**c**) S200_L vs SCK_L

There was a total of 14,059 genes annotated in 140 KEGG pathways in the library of *C. grandiflora* undergoing salt stress for 12 h. Among these genes, differences could be seen in ribosomes, protein processing in the endoplasmic reticulum, plant hormone signal transduction, spliceosomes, and plant pathogen interaction pathways. The number of expressed genes was the most enriched (Fig. 6a). A total of 1417 genes in the root library were annotated in 120 KEGG pathways. Of these genes, phenylpropane biosynthesis, protein processing in the endoplasmic reticulum, starch and sucrose metabolism, plant hormone signal transduction, and galactose metabolism pathways were abundant in DEGs. The number of sets was the largest (Fig. 6b). There was a total of 3480 genes in Ye’s library annotated in 132 KEGG pathways, among which are DEGs in plant pathogen interactions, plant hormone signal transduction, protein processing in the endoplasmic reticulum, starch and sucrose metabolism, and phenylpropane biosynthesis pathways. The number of enrichments was the largest (Fig. 6c).

### Analysis of DEGs of plant salt stress-induced transporters

Although 200 mM NaCl stress for 12 h induced osmotic stress in plants, different ion transporter genes were also modulated, either upregulated or downregulated in the roots and leaves. This is because in order to maintain ion homeostasis and osmotic adjustment under saline conditions, tolerant plants like *C. grandiflora* modify the ion transporters activities to achieve this task [29]. In this study, the root transport genes *HKT1*, *AKT1*, *AKT2*, *NHX2*, *NHX3*, *NHX5*, and *CLC-A* were all upregulated, and *CLC-B* genes were downregulated. In the leaves, the salt stress transporter genes *HKT1*, *CHX17*, *CHX18*, *AKT2*, and *NHX3* were upregulated, and *AKT1*, *CLC-C*, and *CLC-D* were downregulated. We found that the accumulation of Na^+^ in roots was greater than that in leaves after salt stress, while the accumulation of K^+^ in leaves was more, indicating that ion transporter genes could limit the transport of Na^+^ to shoots while maintaining a higher K^+^ content. The expression and regulation of genes related to ion transport such as HKT, AKT and NHX were changed afterward, so that *C. grandiflora*. Showed better osmotic regulation ability and maintained high biofilm stability, thus adapting to the salt stress environment (Table 2).

**Table 2:** Differential genes related to salt stress-induced transporters

Tissue site	Gene_ID	Name	log2FC	regulation
root	TRINITY_DN116960_c1_g1	*HKT1*	0.97	up
	TRINITY_DN119869_c1_g2	*AKT1*	1.05	up
	TRINITY_DN110456_c0_g2	*AKT2*	1.86	up
	TRINITY_DN109744_c0_g1	*NHX2*	0.56	up
	TRINITY_DN90305_c0_g1	*NHX3*	0.50	up
	TRINITY_DN121777_c2_g1	*NHX5*	0.45	up
	TRINITY_DN109895_c0_g3	*CLC-A*	0.78	up
	TRINITY_DN109895_c0_g1	*CLC-B*	−0.63	down
leaf	TRINITY_DN101309_c3_g1	*HKT1*	3.39	up
	TRINITY_DN124753_c1_g3	*CHX17*	3.82	up
	TRINITY_DN124753_c1_g2	*CHX18*	4.38	up
	TRINITY_DN110456_c0_g2	*AKT2*	3.69	up
	TRINITY_DN90305_c0_g1	*NHX3*	2.10	up
	TRINITY_DN124299_c2_g4	*AKT1*	−1.48	down
	TRINITY_DN115730_c1_g1	*CLC-C*	−1.44	down
	TRINITY_DN108068_c1_g3	*CLC-D*	−1.26	down

### Analysis of DEGs in the phenylpropanoid biosynthesis pathway

**Fig. 7:**
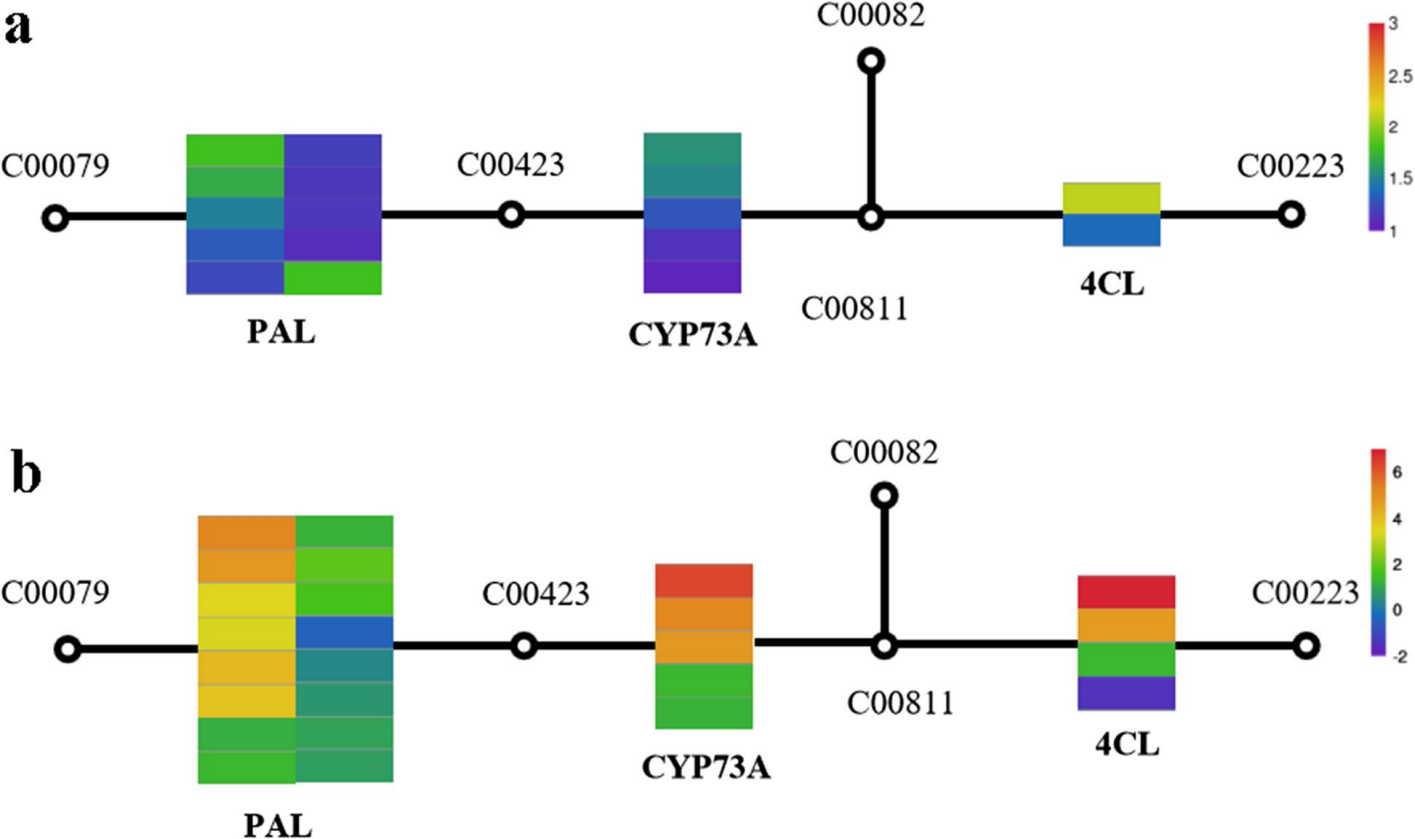
DEGs in the phenylpropane biosynthesis pathway under 200 mM NaCl stress, (**a**) Roots, (**b**) leaves

Phenylpropane compounds play an important role in plant growth, development, and response to adverse stresses. The phenylpropane biosynthesis pathway is important for plant secondary biomass metabolism. All substances containing the phenylpropane skeleton are the direct or indirect products of this pathway, and these compounds play a critical role in the growth, development, and resistance of plants. Phenylpropane biosynthesis also has an important physiological significance in plants, which is mainly manifested by changes in enzyme activity and the differentiation of intermediate products, further transformation products, and cells in plant development. In this study, there were 268 DEGs annotated in the phenylpropanoid biosynthesis pathway in the *C. grandiflora* samples. In the phenylpropane synthesis pathway in the roots, all 17 of the DEGs were upregulated, including *PAL* (10), *CYP73A* (5), and *4CL* (2). On the other hand, in the phenylpropane synthesis pathway in the leaves, there were 25 DEGs, of which 24 were upregulated, including *PAL* (16), *CYP73A* (5), and *4CL* (3). We found that there were more upregulated genes in the leaves, indicating that under conditions of salt stress, phenylpropane biosynthesis in *C. grandiflora* leaves was more active, thereby providing leaves with more salt stress resistance (Table 3, Fig. 7).

**Table 3:** Differentially expressed genes in the phenylpropanoid biosynthesis pathway

Tissue site	Gene_ID	KOEntry	Name	log2FC	regulation
root	TRINITY_DN110915_c2_g1	K10775	PAL	1.69	up
	TRINITY_DN95790_c1_g4	K10775	PAL1	1.65	up
	TRINITY_DN123821_c2_g5	K10775	PAL	1.49	up
	TRINITY_DN112712_c0_g1	K10775	PAL5	1.32	up
	TRINITY_DN101076_c4_g6	K10775	PAL	1.23	up
	TRINITY_DN112712_c0_g3	K10775	PAL1	1.19	up
	TRINITY_DN105957_c0_g1	K10775	PAL1	1.16	up
	TRINITY_DN94239_c0_g3	K10775	PAL1	1.15	up
	TRINITY_DN112712_c0_g2	K10775	PAL	1.10	up
	TRINITY_DN123821_c2_g1	K10775	PAL	1.79	up
	TRINITY_DN93014_c2_g1	K00487	CYP73A12	1.58	up
	TRINITY_DN115534_c1_g1	K00487	CYP73A1	1.54	up
	TRINITY_DN93497_c3_g1	K00487	CYP73A1	1.29	up
	TRINITY_DN115534_c1_g3	K00487	CYP73A1	1.14	up
	TRINITY_DN115534_c0_g1	K00487	CYP73A13	1.07	up
	TRINITY_DN100929_c1_g1	K01904	4CL2	2.12	up
	TRINITY_DN122193_c0_g2	K01904	4CL	1.40	up
leaf	TRINITY_DN123821_c2_g4	K10775	PAL	4.21	up
	TRINITY_DN112712_c0_g2	K10775	PAL	4.05	up
	TRINITY_DN94239_c0_g3	K10775	PAL1	3.73	up
	TRINITY_DN112712_c0_g3	K10775	PAL1	3.61	up
	TRINITY_DN123821_c2_g5	K10775	PAL	3.41	up
	TRINITY_DN123821_c2_g1	K10775	PAL	3.35	up
	TRINITY_DN110915_c2_g1	K10775	PAL	2.74	up
	TRINITY_DN94239_c0_g4	K10775	PAL	2.63	up
	TRINITY_DN95790_c1_g1	K10775	PAL	2.50	up
	TRINITY_DN94239_c0_g1	K10775	PAL1	2.49	up
	TRINITY_DN123821_c1_g1	K10775	PAL1	2.45	up
	TRINITY_DN94239_c0_g5	K10775	PAL	2.32	up
	TRINITY_DN105957_c0_g1	K10775	PAL1	2.28	up
	TRINITY_DN110915_c2_g3	K10775	PAL	2.20	up
	TRINITY_DN123821_c1_g2	K10775	PAL	2.07	up
	TRINITY_DN108603_c1_g2	K10775	PAL	1.71	up
	TRINITY_DN115534_c0_g1	K00487	CYP73A13	6.47	up
	TRINITY_DN93497_c3_g1	K00487	CYP73A1	5.62	up
	TRINITY_DN115534_c1_g1	K00487	CYP73A1	5.36	up
	TRINITY_DN115534_c1_g3	K00487	CYP73A1	2.63	up
	TRINITY_DN93014_c2_g1	K00487	CYP73A12	2.55	up
	TRINITY_DN100199_c0_g1	K01904	4CL9	1.39	up
	TRINITY_DN100929_c1_g1	K01904	4CL2	6.93	up
	TRINITY_DN122193_c0_g2	K01904	4CL	4.78	up

### Analysis of DEGs in the plant hormone signal transduction pathway

**Fig. 8:**
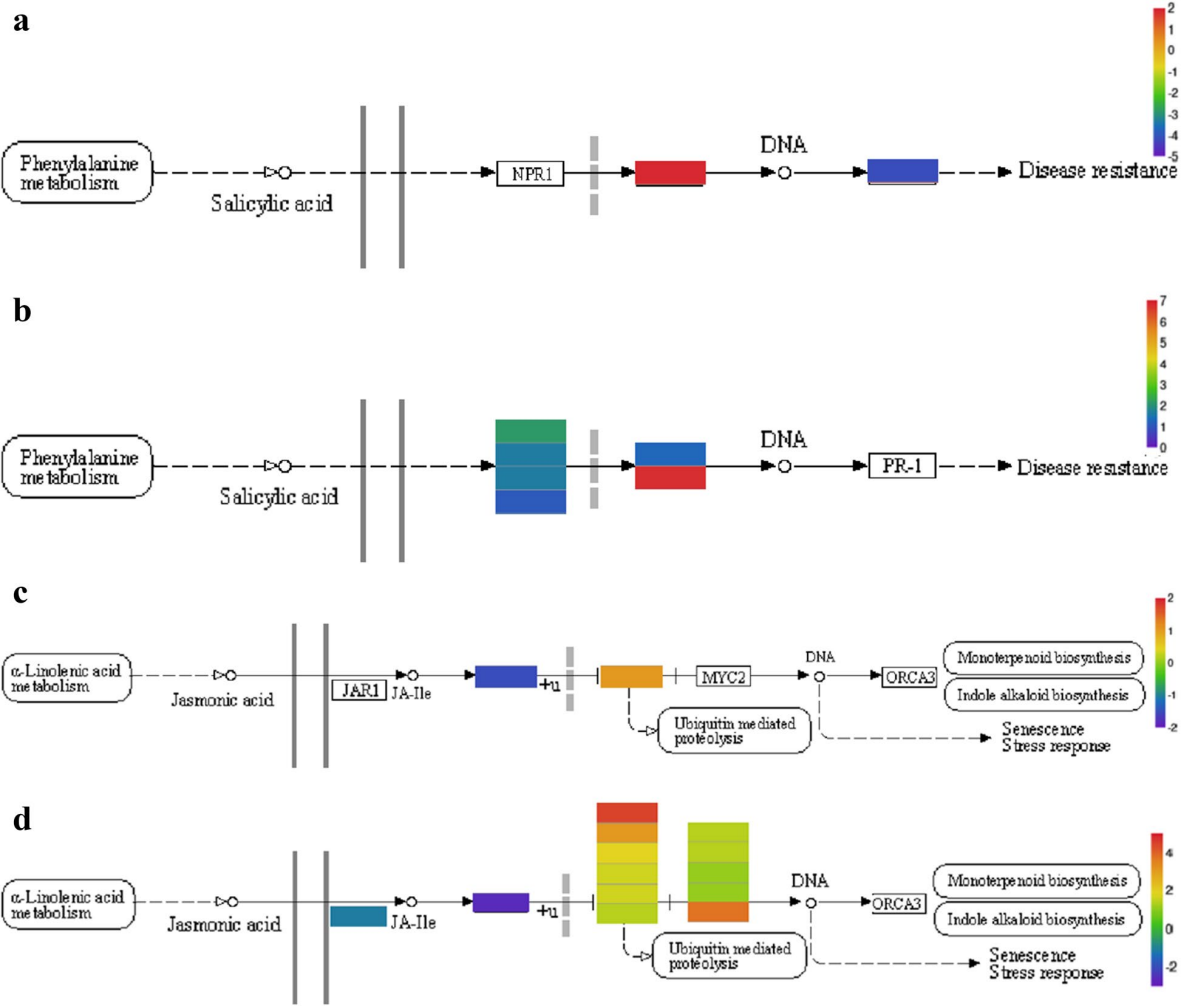
The salicylic acid and jasmonic acid signal transduction pathways: salicylic acid (**a**) roots, (**b**) leaves; jasmonic acid (**c**) roots, (**d**) leaves

Phytohormones are key endogenous factors that mediate plant stress responses, and they play an important role in plant defense and response to environmental stimuli. In the plant hormone signal transduction pathways of this study, a total of 329 DEGs were found. We focused on the salicylic acid and jasmonic acid signal transduction pathways. The results showed that there were 7 DEGs in the salicylic acid signal transduction pathway in the *C. grandiflora* samples, of which 6 genes were upregulated and 1 gene was downregulated; there were 13 DEGs in the jasmonic acid signal transduction pathway, of which 11 genes were upregulated and 2 genes were downregulated. In the leaves and roots, both signal transduction pathways revealed DEGs that were annotated. *TGA7* was upregulated and *PR1B1* was downregulated in the salicylic acid signal transduction pathway in the roots. In the jasmonic acid signal transduction pathway, *TIFY9* was upregulated, and *COI2* was downregulated in the roots. *NPR1*, *NPR2*, *NPR3*, *TGAL5*, and *TGA7* in the salicylic acid signal transduction pathway in the leaves were all upregulated. Similarly, *TIFY10B*, *TIFY9*, *TIFY10A*, *TIFY6A*, *TIFY6B*, *MYC2*, *MYC4* and *AIB* were all upregulated in the jasmonic acid signal transduction pathway in the leaves, yet *GH3.5* and *COI* were downregulated. By comparing the expression patterns of DEGs in the roots and leaves, we found that the number of leaf genes in both the salicylic and jasmonic acid signal transduction pathway was relatively larger than roots. The differential gene expression patterns of the salicylic and jasmonic acid signal transduction pathways in the leaves and roots were similar (Table 4, Fig. 8).

**Table 4:** Differentially expressed genes of the plant hormone signal transduction pathways

Tissue site	Hormone signal transduction pathway	Gene_ID	KOEntry	Name	log2FC	regulation
root	Salicylic acid signal transduction pathway	TRINITY_DN104980_c2_g1	K14431	*TGA7*	1.88	up
		TRINITY_DN87692_c0_g1	K13449	*PR1B1*	−4.10	down
	Jasmonic acid signal transduction pathway	TRINITY_DN111903_c2_g1	K13463	*COI2*	−1.48	down
		TRINITY_DN91100_c0_g2	K13464	*TIFY9*	1.05	up
leaf	Salicylic acid signal transduction pathway	TRINITY_DN93638_c0_g1	K14508	*NPR3*	2.14	up
		TRINITY_DN111453_c0_g1	K14508	*NPR2*	1.69	up
		TRINITY_DN107526_c3_g1	K14508	*NPR3*	1.68	up
		TRINITY_DN123194_c1_g2	K14508	*NPR1*	1.11	up
		TRINITY_DN119181_c0_g2	K14431	*TGAL5*	1.31	up
		TRINITY_DN104980_c2_g1	K14431	*TGA7*	6.81	up
	Jasmonic acid signal transduction pathway	TRINITY_DN123106_c1_g2	K14506	*GH3.5*	−1.11	down
		TRINITY_DN111903_c2_g1	K13463	*COI2*	−2.60	down
		TRINITY_DN110816_c0_g1	K13464	*TIFY10B*	4.47	up
		TRINITY_DN91100_c0_g2	K13464	*TIFY9*	3.07	up
		TRINITY_DN92901_c0_g2	K13464	*TIFY10A*	1.91	up
		TRINITY_DN91844_c0_g3	K13464	*TIFY6A*	1.67	up
		TRINITY_DN116306_c1_g1	K13464	*TIFY10A*	1.63	up
		TRINITY_DN113060_c0_g1	K13464	*TIFY6B*	1.37	up
		TRINITY_DN109853_c2_g3	K13422	*MYC2*	1.44	up
		TRINITY_DN111782_c0_g3	K13422	*MYC4*	1.42	up
		TRINITY_DN120917_c6_g4	K13422	*MYC4*	1.11	up
		TRINITY_DN111782_c0_g1	K13422	*AIB*	1.08	up
		TRINITY_DN110997_c0_g2	K13422	*BHLH14*	3.76	up

### Differentially expressed transcription factor analysis

**Fig. 9:**
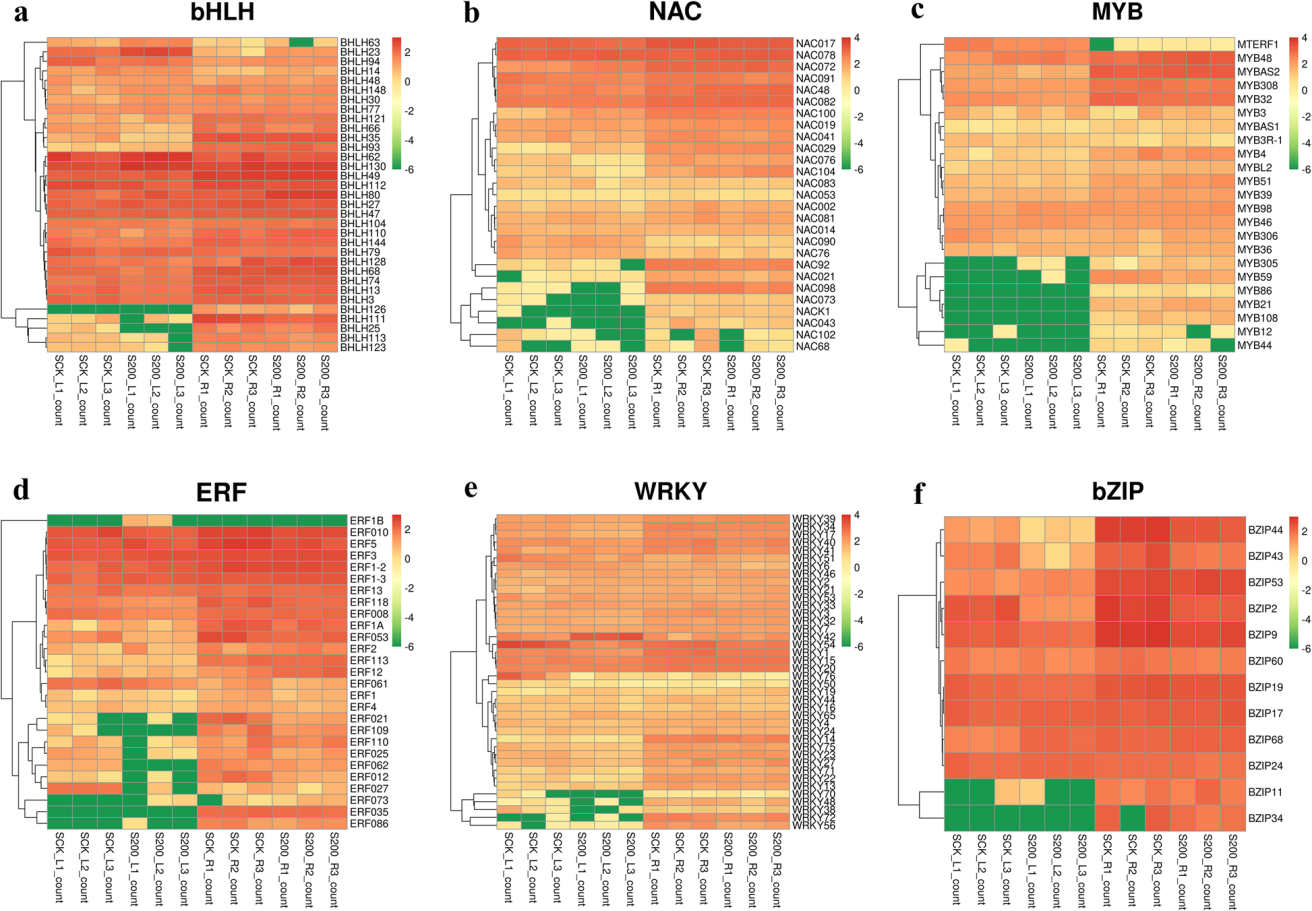
Heat map of transcription factor family expression

As transacting factors, transcription factors can bind to cis-acting element regions upstream of the target gene through the protein structure specific DNA binding region, thereby activating the expression of the target gene. This transcription level regulation is the most important way to regulate gene expression. From a protein structure analysis, a transcription factor is composed of a DNA binding region, a transcription regulatory domain, an oligomerization site, and a nuclear localization signal. These functional regions determine a transcription factor structure and characteristics. According to the characteristics of DNA binding regions, transcription factors can be divided into different families. In this study, a total of 1604 transcription factors from 54 families were annotated. Among them, the transcription factor families bHLH, NAC, MYB, ERF, WRKY, and bZIP had the most annotated transcription factors, which were 171, 139, 101, 88, 82, and 75, respectively (Table 5, Fig. 9).

**Table 5:** Analysis of differentially expressed transcription factors

TF_family	TF_ID	Quantity	Percentage
bHLH	MDP0000254650	171	10.66%
NAC	KN539001.1_FGP003	139	8.67%
MYB	Aan017619	101	6.30%
ERF	GSVIVT01018226001	88	5.49%
WRKY	Aan015851	82	5.11%
bZIP	Do001279.1	75	4.68%
FAR1	KN538871.1_FGP005	74	4.61%
GRAS	MDP0000258655	68	4.23%
C3H	Bostr.18994 s0001.1.p	64	4.00%
B3	676,753,378	58	3.61%
C2H2	MDP0000321222	58	3.61%
G2-like	KHN04400.1	47	2.93%
MYB	Sme2.5_02552.1_g00001.1	44	2.74%
GATA	KN538788.1_FGP023	41	2.56%

### Analysis of gene expression with qRT-PCR

We selected 16 candidate DEGs for transcription polymerase chain reaction (qRT-PCR) analysis to verify the accuracy of the Illumina sequencing data. In the two processing parts, qRT-PCR and RNA-seq analysis showed that the single gene expression trend was basically the same (Fig. S3). The gene expression results revealed that the transcriptome sequencing data reflected the response of the roots and leaves of the *C. grandiflora* to NaCl stress.

## Discussion

Under saline conditions, plants utilize a series of physiological and biochemical responses to cope with the stressful effects [30]. Excessive salt in the growth medium induces osmotic stress and causes plant cells to lose water, while long term salinity stress causes nutrient imbalance and ion toxicity [31]. Salinity-induced oxidative stress is a secondary stress due to the increase in reactive oxygen species that leads to metabolic disorders and a decline in photosynthetic efficiency [32]. Excessive amounts of active oxygen leading to an excessive membrane peroxidation reaction, which produces harmful substances like malondiene. In order to cope with the increase in active oxygen, plants own peroxidation protection enzyme systems (e.g., SOD, POD, and CAT) [13]. In this study, the tissue water content of *C. grandiflora* showed a downward trend, which indicates a water deficit induction by NaCl imposition at short term salinity, and ionic effect at long term salinity [10]. Osmotic stress reduces the ability of plants to absorb water; this phase is short-lived resulting in stomatal closure and inhibition of cell expansion in the bud [10, 32]. In response to osmotic stress, tolerant plants synthesize and accumulate compatible osmolytes and/or inorganic ions (provided they are compartmentalized into vacuoles to reduce their cytotoxicity) in order to maintain water absorption and turgor pressure, thereby alleviate plant growth inhibition [33–35]. In the present study, the accumulation of Na^+^ in *C. grandiflora* may contribute to osmotic adjustment, thereby allowing *C. grandiflora* to adapt to the salinity stress.

The enzyme activities of SOD, POD, and CAT of *C. grandiflora* initially increased followed by a subsequent decrease with time and with increasing salt concentrations. This response might indicate that after long time and high concentration of salt imposition, antioxidant enzymes’ effectiveness was greatly inhibited to scavenge excessive reactive oxygen species. Alternatively, non-enzymatic antioxidants may increase and take a role to eliminate the hazardous impact of ROS [36].

Studies have shown that under salt stress, Na^+^ enters the cell through nonselective cation channels and causes plasma membrane depolarization, which in turn activates the outward K^+^ channel, allowing K^+^ to flow out of the cell [37]. In this study, with an increase in time under salt stress, the Na^+^ content gradually increased and the K^+^ content gradually decreased in the roots and leaves of *C. grandiflora*, which agrees with the previous studies. There are a large number of ion transporter genes that play important roles in the process of ion transport in plants such as *HKT*, *NHX* and *AKT* [38]. During Na^+^ transport in plants, *HKT* is located on the plasma membrane while *NHX* is located on the vacuolar membrane [39]. *HKT* is responsible for the recovery of Na^+^ entering the root xylem into xylem parenchyma cells, thereby reducing the Na^+^ accumulation of xylem. *NHX* sequesters Na^+^ into the vacuoles and thus reducing its ion toxicity for the cytoplasm and other organelles. It is reported that accumulation of Na^+^ in the vacuoles not only avoid cytoplasmic toxicity, but also is used as an osmolyte to alleviate osmotic stress [40]. Our results revealed that there were more upregulated ion transporter genes in the roots, and we concluded that this upregulation of ion transporter genes is one of the important factors that affects *C. grandiflora* response and tolerance to salt stress.

According to GO annotations, the largest number of differentially expressed genes were enriched in the nuclear, plasma membrane, cytoplasm, membrane components, and protein binding in the root library. The largest number of differentially expressed genes were enriched in the leaf library for the nuclear, plasma membrane, cytoplasm, membrane components, and in the chloroplasts. We found that the GO annotations with the highest degree of enrichment of DEGs in the roots and leaves were the same. According to the KEGG annotation, in the samples of *C. grandiflora*, the number of DEGs enriched in the ribosomes and spliceosomes or those genes involved in protein processing in the endoplasmic reticulum, phytohormone signal transduction, and phytopathogen interaction pathways was the largest. We also found the largest number of differentially expressed genes were enriched in the root library for phenylpropane biosynthesis, ER protein processing, starch and sucrose metabolism, plant hormone signaling, and galactose metabolism pathways. Also, the largest number of differentially expressed genes were enriched in the library of plant pathogen interactions, plant hormone signaling, protein processing in the endoplasmic reticulum, starch and sucrose metabolism, and phenylpropane biosynthesis pathways. Transcriptomic and metabolomic analysis have shown that DEGs and differential metabolites obtained in the phenylpropanoid biosynthesis pathway are significantly related under salt stress [41]. Previous studies have found that plant hormones are small chemicals that play a key role in plant growth and development [42]. Stress hormones such as salicylic acid (SA) and jasmonic acid (JA) mediate the balance between salt stress signals and control growth and stress responses. Therefore, we mainly identified the SA and JAs signal transduction pathways in the phenylpropane biosynthesis pathway and the plant hormone signal transduction pathway.

The metabolic pathway of phenylpropane in plants is a very complex metabolic network, which is the main synthesis pathway of important secondary metabolites such as phenols, flavonoids, anthocyanins, and lignins [43]. These secondary metabolites are widely involved in various physiological activities of plants, especially in the response process of plants to biotic and abiotic stresses [44]. In this study, 268 DEGs were annotated in the phenylpropanoid biosynthesis pathway in the *C. grandiflora* samples. Among them, there were 24 DEGs in the phenylpropane synthesis pathway of the leaves, all of which were upregulated, including *PAL*, *CYP73A* and *4CL*. In the roots, there were 17 DEGs in the phenylpropane synthesis pathway, all of which were upregulated including *PAL*, *CYP73A* and *4CL*. *PAL* is the key rate-limiting enzyme that plays an important role in the plant growth and development, connects plant primary and secondary metabolism, and catalyzes the first reaction of phenylpropane metabolism [45]. In addition, PAL is closely related to the content of secondary metabolites (such as lignin, phytoalexin, flavonoids), and it also plays an important role in the response of plants to biotic and abiotic stress [46]. 4-coumarate:coenzyme A ligase (*4CL*) is the last key enzyme for the phenylpropane biosynthesis pathway to shift to the downstream branch pathway. It contains cinnamic acid, 4-coumaric acid, caffeic acid, erucic acid, ferulic acid, and 5-hydroxyferulic acid, which are used as substrates to generate corresponding acyl-CoA esters. Chlorogenic acid is then generated under the action of hydroxycinnamoyl coenzyme quinic acid hydroxycinnamate acyltransferase (*HQT*) [47]. We found that the upregulated expression of genes was closely related to salt tolerance of *C. grandiflora*: upregulated expression of *PAL* in the roots and leaves could affect the content of secondary metabolites, and expression of *4CL* affects chlorogenic acid. The synthesis of chlorogenic acid is closely related to antioxidants [48]. There were more *PAL*, *CYP73A* and *4CL* genes involved in phenylpropane biosynthesis in the leaves of *C. grandiflora*. Therefore, we speculate that the phenylpropane biosynthesis pathway is an important tolerance feature for leaves to defend against salt stress.

Plants use complex signaling pathways to respond to stress. In addition to some other small molecules (such as Ca^2+^ and ROS), plant hormones can initiate a specific signal cascade after sensing biological and abiotic stress [49]. Fluctuations in the levels of several major hormones such as ABA, ET, SA, and JA, as an early response to stress, initiate metabolic processes that lead to changes in the plant growth patterns. The Nonexpressor of Pathogenesis-Related gene 1 (*NPR1*) is an activator of plant resistance. *NPR1* not only plays a core regulatory role in plant system resistance acquisition and induction of systemic resistance, but also acts as an important regulatory factor for plant basic resistance and resistance determined by disease resistance genes [50]. Transcription factors TGA [51], TIFY [52], and MYC [53] also play an important role in plant resistance to abiotic stresses. This study revealed that a large number of genes related to salt stress were in the signal transduction pathway of plant hormones, and the number of DEGs in the leaves was relatively large. In the SA signal transduction pathway, *NPR1*, *NPR2*, *NPR3*, *TGAL5* and *TGA7* are upregulated, and *TGA7* was upregulated in the roots and leaves; the rest were only upregulated in leaves. *TIFY10B*, *TIFY9*, *TIFY10A*, *TIFY6A*, *TIFY6B*, *MYC2*, *MYC4* and *AIB* are upregulated in the JA signal transduction pathway. Studies have shown that signal recognition is a key step for JA to play a role in plants, and TIFY is a key factor that regulates the signal recognition of JA [54]. When plants activate the JAs signal pathway, *MYC2*, *bHLH* and other transcription factors are inhibited by the TIFY protein through ubiquitination [55]. The first step toward activation of this pathway begins when the content of exogenous or endogenous JA increases. With this increase in JA, the JA receptor *COI1* binds to the TIFY protein to form a *SCFCOI1* complex. The deubiquitinated TIFY protein is degraded by the 26S proteasome, and the *MYC2* and *bHLH* transcription factors are released concurrently, which finally activates the JAs signal pathway. Since *TIFY9* was upregulated in the roots and leaves in this study, by comparing gene expression patterns, we found that there were more upregulated genes in the leaves. Therefore, we speculated that *TIFY9* in *C. grandiflora* could play a role in both the roots and the leaves. It is clear that both SA the JA signal transduction pathways are important mechanisms to defend against salt stress in the leaves of *C. grandiflora*.

Plant transcription factors are members of a vital gene family that are widely present and participate in the regulation of plant growth, development and resistance to stresses. Transcription factors can regulate the expression of stress-responsive genes by combining with specific action elements in downstream gene promoters, thereby directly or indirectly participating in many biological processes of plants, such as cell morphogenesis, metabolism, physiological balance, and signal transduction [56]. Studies have shown that transcription factor families such as bHLH, NAC, MYB, ERF, WRKY, and bZIP regulate several aspects of plant growth, development, secondary metabolism, and stress response [57–60]. In this study, the transcription factor families bHLH, NAC, MYB, ERF, WRKY and bZIP had the most annotated transcription factors, which suggests that these transcription factor families are strongly participate in salinity tolerance of *C. grandiflora*.

**Fig. 10:**
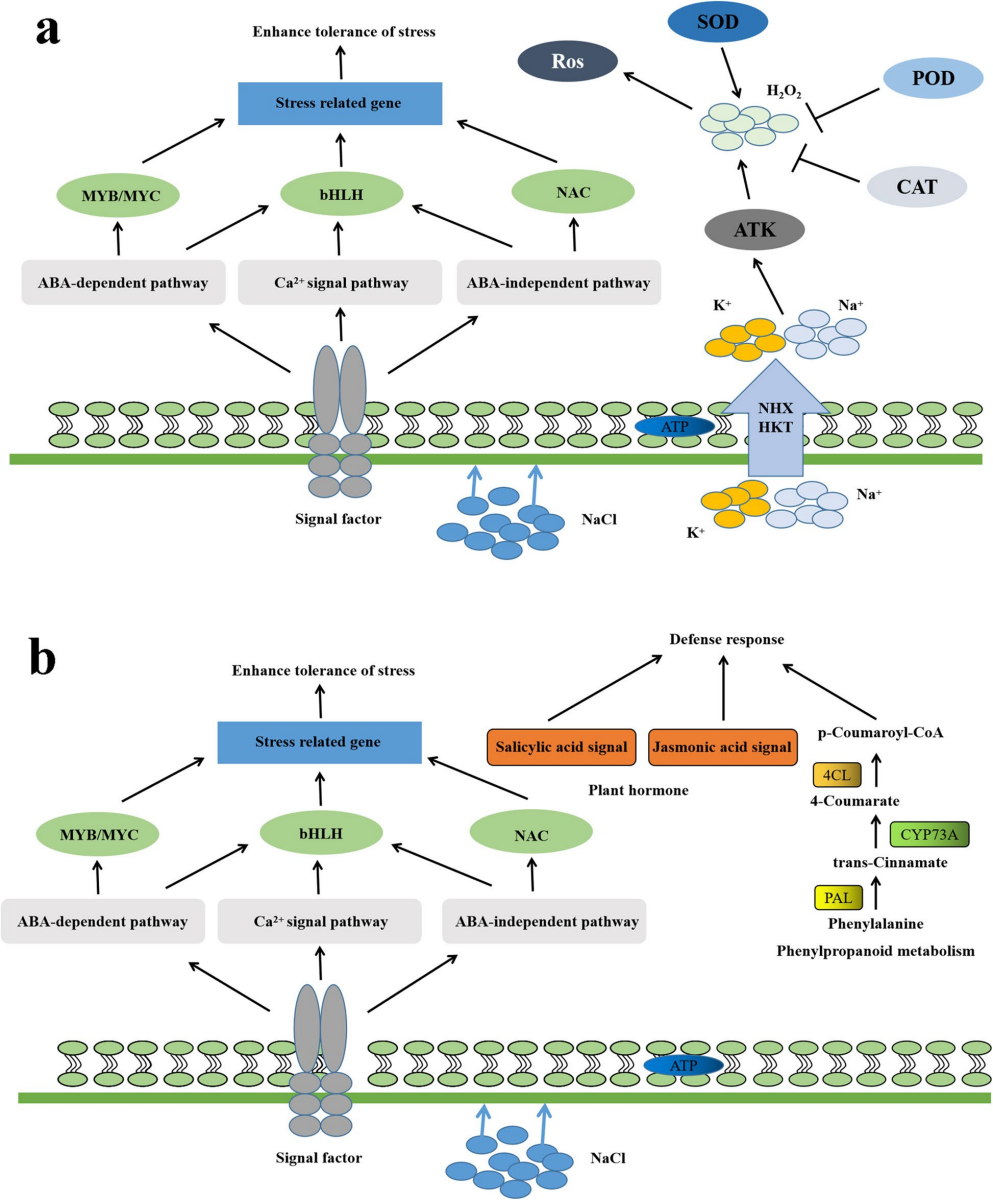
Molecular mechanism diagram of salt stress resistance of *C. grandiflora*: (**a**) roots, (**b**) leaves

In summary, transcriptome sequencing showed that salt stress changed the transcription levels of many genes related to multiple regulatory networks, including osmotic regulation, ion transport, reactive oxygen scavenging, and plant hormone signal regulation. Studies demonstrated that there are more DEGs in the roots of the chrysanthemum cv. ‘Jinba’ than in the leaves, and the number of downregulated genes involved in the salt stress response exceeds the number of upregulated genes under salt stress [12]. In addition, the roots of chrysanthemums under salt stress are mainly dominated by the upregulation of genes encoding proteins involved in ion transport, while in the leaves the response is focused on osmotic regulation and Ca^2+^ transport [11]. Our research showed that there were more DEGs in the leaves of *C. grandiflora* than in its roots, and more upregulated than downregulated genes in the roots and leaves in response to salt stress. Salinity responses in root include upregulations of genes encoding ionic transporters, thereby changing the osmotic regulation ability of *C. grandiflora*. Contrary, the leaf response focused on the SA and JAs signal transduction pathways in phenylpropane biosynthesis and plant hormone signal transduction. We propose that this may be a result of the differences in the resistance of plant materials. Similarly, drought and salt stress on *Chrysopogon zizanioides* resulted in more transcriptome changes in leaf tissues than in root tissues [13]. The results of the previous work are similar to our findings concerns the number of genes and their expression patterns in the roots and leaves of *C. grandiflora*. Figure 10 shows a molecular mechanism diagram of *C. grandiflora* that summarizes *C. grandiflora* response and tolerance mechanism under saline conditions.

## Conclusions

The results of transcriptome sequencing showed that salt stress changed the transcription levels of many genes related to multiple regulatory networks, including osmotic regulation, ion transport, and reactive oxygen scavenging systems. The functional enrichment analysis of candidate genes showed that the tissue-specific pattern of the transcriptome under salt treatment: in *C. grandiflora*, more transcriptome changes and number of genes involved in the salt stress response was greater in the leaf tissues than the roots. Root response to salinity included upregulation of genes encoding involved ion transporters. However, the leaf response focused on phenylpropane biosynthesis and plant hormone signal transduction. This work has greatly enriched the existing sequence resources of *C. grandiflora* and provides a large number of salt-tolerant candidate genes for further functional analysis to improve plant salt tolerance.

## Supplementary Information

**Additional file 1.**

## Data Availability

The datasets supporting the conclusions of this article are included within the article. Sequencing database for *Chrysanthemum*×*grandiflora* could download from NCBI under the accession number SRR17510868, SRR17510867, SRR17510866, SRR17510865, SRR17510874, SRR17510873, SRR17510876, SRR17510875, SRR17510872, SRR17510871, SRR17510870 and SRR17510869. The data will be shared on reasonable request of the corresponding author.
