# Supplementary material for: Chrysanthemum × grandiflora leaf and root transcript profiling in response to salinity stress
Source: BMC Plant Biol. 2022 May 12;22:240. doi: 10.1186/s12870-022-03612-x (PMC9097105; doi:10.1186/s12870-022-03612-x)
Supplement: Supplementary file 1 — Additional file 1. [file 12870_2022_3612_MOESM1_ESM.docx]

**Supplementary materials**

**Table S1** Statistics of output sequence of *C. Grandiflora*

| Samples | Raw_Reads (No.) | Valid_reads (No.) | Raw_Bases (bp) | Valid_Bases (bp) | Q20 (%) | Q30 (%) |
| --- | --- | --- | --- | --- | --- | --- |
| SCK_L1 | 43,153,100 | 42,326,634 | 6.47G | 5.90G | 97.84 | 93.34 |
| SCK_L2 | 46,156,038 | 44,286,876 | 6.92G | 6.13G | 97.34 | 92.45 |
| SCK_L3 | 41,415,656 | 39,998,304 | 6.21G | 5.54G | 97.43 | 92.68 |
| S200_L1 | 49,751,488 | 49,026,640 | 7.46G | 6.80G | 97.41 | 92.24 |
| S200_L2 | 41,337,408 | 39,900,828 | 6.20G | 5.53G | 97.49 | 92.68 |
| S200_L3 | 43,824,844 | 42,081,102 | 6.57G | 5.82G | 97.31 | 92.46 |
| SCK_R1 | 47,160,892 | 45,560780 | 7.07G | 6.31G | 97.35 | 92.38 |
| SCK_R2 | 49,676,308 | 47,754,498 | 7.45G | 6.60G | 97.05 | 91.93 |
| SCK_R3 | 50,919,822 | 50,130,238 | 7.64G | 6.95G | 97.64 | 93.02 |
| S200_R1 | 50,235,962 | 49,426,242 | 7.54G | 6.83G | 97.46 | 92.76 |
| S200_R2 | 46,372,458 | 45,630,372 | 6.96G | 6.30G | 97.24 | 92.39 |
| S200_R3 | 48,050,494 | 47,205,962 | 7.21G | 6.50G | 97.18 | 92.38 |

**Table S2** Annotation results of *C. Grandiflora* in the database

| Annotation databases | Number of genes | Percentage (%) |
| --- | --- | --- |
| NR | 31181 | 79.57 |
| GO | 25624 | 65.39 |
| KEGG | 20601 | 52.57 |
| eggNOG | 27484 | 70.14 |
| Swiss-prot | 21333 | 54.44 |
| All | 39185 | 100.00 |

**
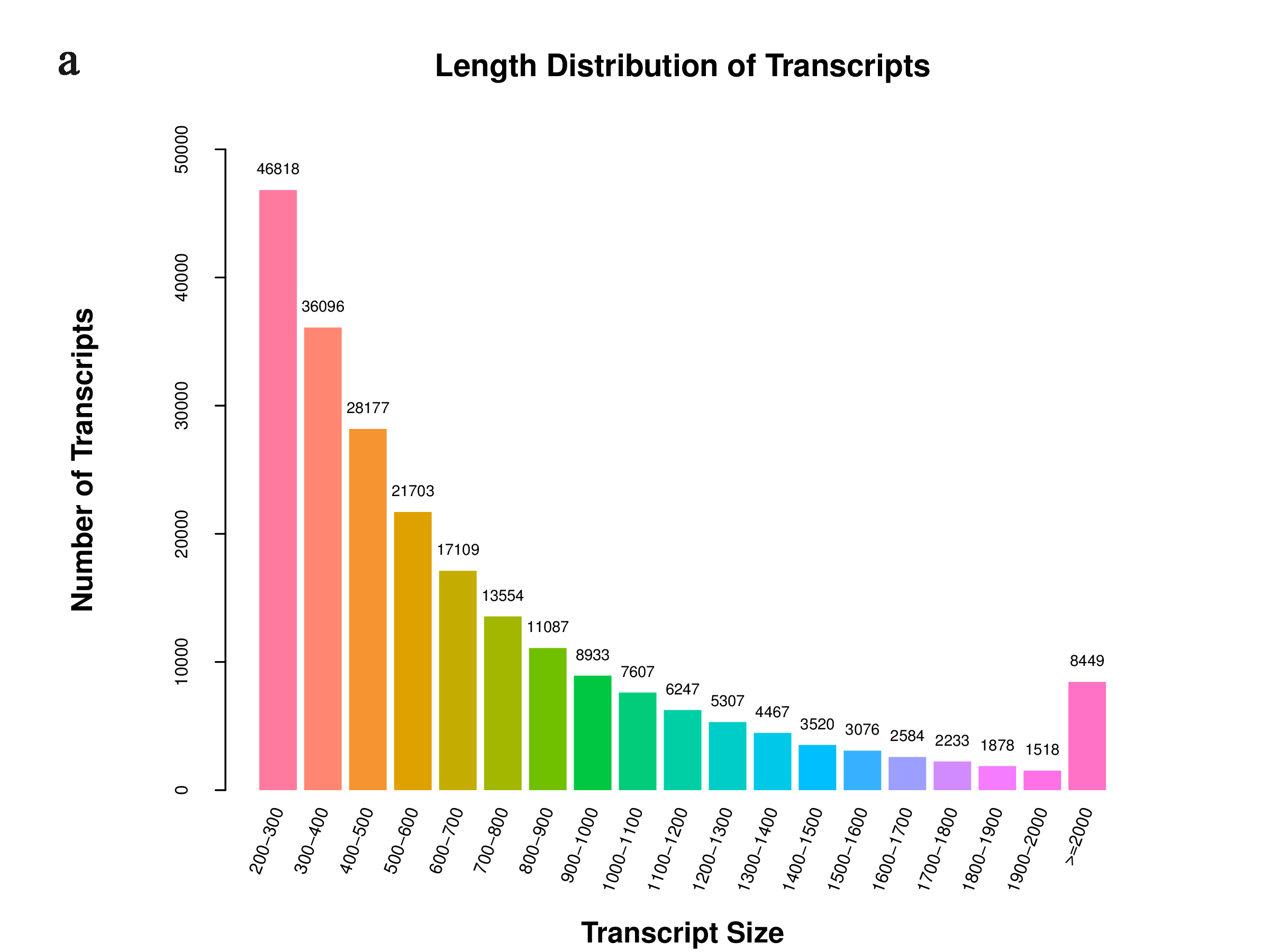

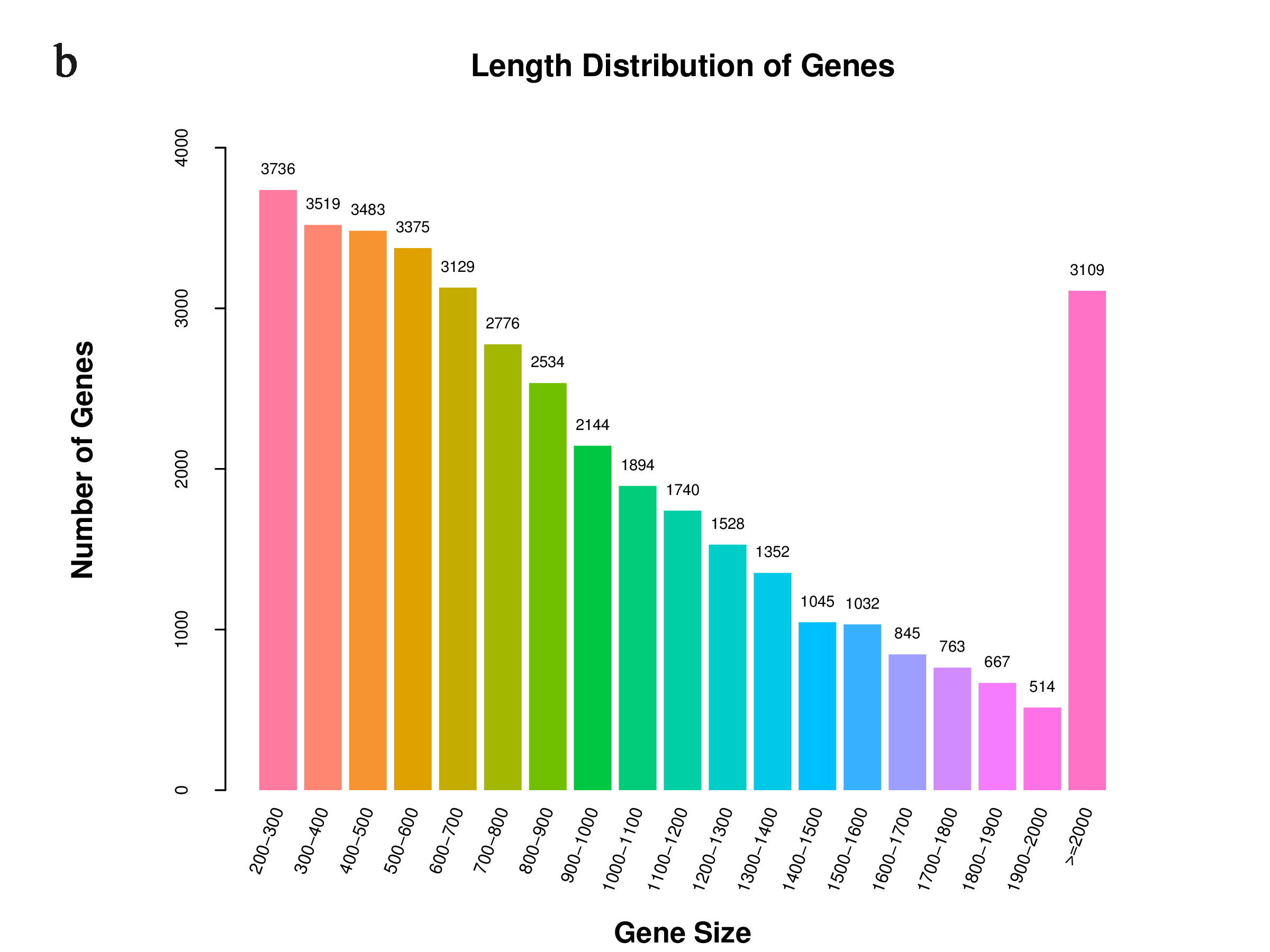
**

**Figure S1** The length distribution of transcript (a) and single genes (b) in *C. Grandiflora*.

**
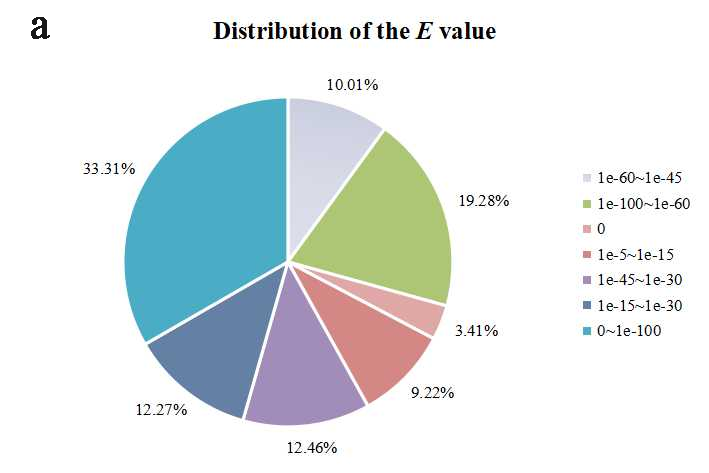

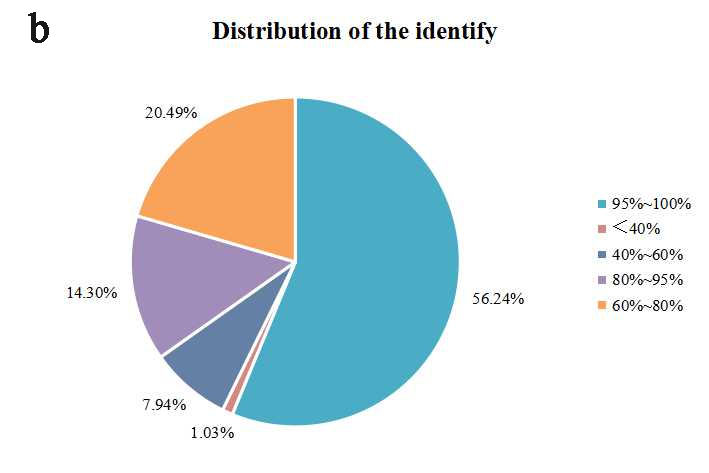

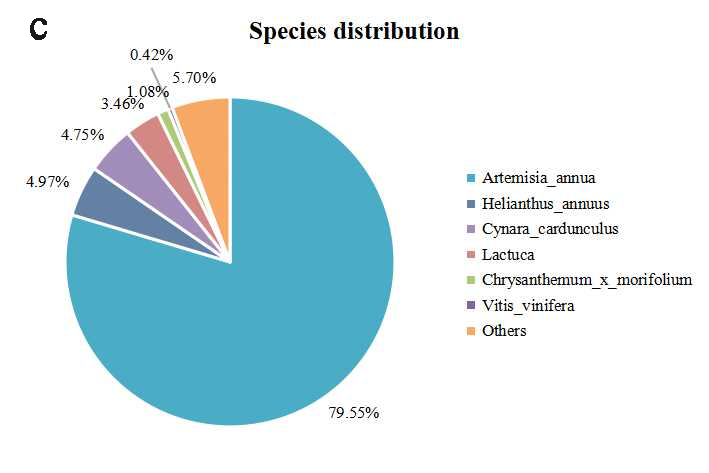
**

**Figure S2** The homology comparison of the single gene in *C. Grandiflora* with the NR database. (a) Distribution of *E* values in the results annotated in the NR database. (b) Single gene distribution identification of *C. Grandiflora*. (c) Distribution of species with homology to *C. Grandiflora*.

**Figure S3** The 16 candidate genes in the *C. Grandiflora* were identified by RNA-seq and qRT-PCR to determine their multiple changes. The *x*-axis represents the treatment site and the control site, and the *y*-axis represents the multiple of change in log_2_FC.
